# Ovarian neoplasms — the role of minimally invasive surgery

**DOI:** 10.3332/ecancer.2025.2030

**Published:** 2025-11-13

**Authors:** Alyssa Stetson, Roshni Dasgupta

**Affiliations:** Division of Paediatric General and Thoracic Surgery, Cincinnati Children Hospital Medical Center, University of Cincinnati, Cincinnati OH 45229, USA

**Keywords:** ovarian neoplasm, paediatric surgery, paediatric gynecology, minimally invasive surgery

## Abstract

The role of minimally invasive surgery (MIS) for ovarian neoplasms in paediatric patients depends on multiple factors. First, it is important to consider the risk of malignancy, which can be difficult to assess, especially in the setting of torsion. Second, when possible ovarian sparing surgery (OSS) should be performed. In certain settings MIS to perform OSS may carry a higher risk of tumour or cyst spillage. MIS can also play a role in diagnosis, via staging or biopsy. When performed, MIS can offer improved visualization of the contralateral ovary and other abdominal structures. Overall, MIS for ovarian neoplasms offers improved visualization of pelvic structures and decreased risk of adhesions in addition to the traditional benefits of MIS. However, these advantages should not supersede the need to achieve complete oncologic resection and to minimize the risk of capsule rupture.

## Introduction

Ovarian neoplasms in paediatric patients present a unique challenge due to the need to balance ovarian preservation with complete resection of malignancy. Patients with benign ovarian masses should undergo ovarian sparing surgery (OSS), but failure to perform an oncologic resection of a malignant tumour can result in the need for additional surgery and adjuvant therapy, and an increased chance of recurrence [1, 2]. Adding to the challenge, it can be difficult to standardise care as a paediatric patient with an ovarian mass may be cared for by a variety of specialists (i.e., paediatric surgeons, paediatric and adolescent gynecologists and adult gynecologists) [3]. Finally, children with ovarian malignancies may present urgently or emergently with torsion, which can limit the extent of workup prior to surgery [4].

Choosing to take an open or minimally invasive surgery (MIS) approach depends on the likelihood of malignancy, yet unfortunately, this can be difficult to characterise. The vast majority (75%–97%) of ovarian masses in children are benign [5–9]. Various studies have sought to discover a method to identify those that are malignant. While malignant tumours are usually larger, the cutoff at which a mass is almost certainly malignant spans a wide range from 5 to 10 cm, and additionally, some studies have found no difference in size between benign and malignant masses [2, 10–12]. Malignancies are more likely to have large (≥ 2–3 mm) septations and nonhyperechoic solid nodular or papillary components, but these features are also not fully predictive, with up to 16% of cystic-appearing masses ultimately malignant [5, 10]. A panel of tumour markers is the most sensitive and specific manner to identify malignancy, but even still, tumour markers may be elevated in 20% of benign masses, especially in the setting of torsion. Additionally, if a patient presents with torsion, it may not be possible to wait for these laboratory tests to result [2, 11]. If time permits, pathology/molecular biology may be used to assist in diagnosis and guide decision-making strategies [13]. The technical approach also depends on the surgeon comfort, with some areas of the world having limited access to resources and training that are necessary to safely perform laparoscopic surgery [14]. This can be further compounded by delays in presentation secondary to long transportation distances and/or long wait times to see a surgeon [14].

OSS is recommended for benign ovarian masses due to its numerous benefits compared to oophorectomy: it decreases the risk of early menopause secondary to premature ovarian failure, offers improved sexual health and higher success with ovarian stimulation for assisted reproduction and avoids low bone density, neurologic disease and cardiac disease in adulthood [5, 15]. Laparoscopic OSS has low rates of conversion to open, and when compared to open surgery, the operative time, estimated blood loss, haemoglobin drop postoperatively, length of stay and complication rates are equivalent or superior [5, 7, 16, 17]. Furthermore, in addition to the other well-documented benefits of MIS surgery (improved cosmesis, decreased pain, shorter length of stay and reduced perioperative morbidity), MIS causes fewer pelvic adhesions which can contribute to improved fertility [16, 18].

## Indications for MIS

### Benign disease

Procedures for benign disease include cyst aspiration, fulguration and OSS. A laparoscopic approach is well established for cyst aspiration and fulguration [16]. More attention has been paid to the outcomes of laparoscopic OSS.

One main concern with laparoscopic OSS is tumour spillage, which can carry the risk for chemical peritonitis for dermoid tumours. While some studies have identified a higher risk of tumour spillage with MIS approach, others have found equivalent rates of spillage regardless of whether the surgery was performed via open or laparoscopy, with increased rates of spillage based only on whether the procedure was OSS versus oophorectomy [7]. Additionally, given the rarity of chemical peritonitis secondary to dermoid spillage, a laparoscopic approach remains reasonable [17, 19]. Conversion rates, which are approximately 5%–10%, do not indicate a high likelihood of failure to complete the procedure via an MIS approach for technical reasons [7, 20].

Another concern with a laparoscopic OSS approach is the risk of cyst or tumour recurrence. However, multiple studies have found either equivalent rates of cystic rupture between open and laparoscopic approaches or equivalent rates of recurrence, even if there is a higher rate of rupture with laparoscopic surgery [21, 22]. Likewise, there does not appear to be higher rates of solid tumour recurrence with MIS [19, 20]**.**

Finally, MIS does not affect ovarian viability, with equivalent blood flow and follicle presence seen after MIS as with open surgery [17, 19].

### Malignant disease

There are several indications for MIS for malignant ovarian neoplasms. Diagnostic laparoscopy can be used to identify the precise location and nature of the neoplasm, including taking a biopsy if indicated [1]. Diagnostic laparoscopy also provides an opportunity for staging, with superior visualisation of the sus-mesocolic and anterior parietal peritoneum as compared to open surgery, as well as easy inspection of the contralateral ovary and diaphragm [1, 23]. In adults, laparoscopy has also been used for debulking advanced cancer and assessing response to neoadjuvant chemotherapy [18, 24, 25]. However, literature is limited in the paediatric population and so there is currently no data on whether a laparoscopic approach should be taken for this indication in children.

### Emergency/unknown malignancy status

Ovarian torsion is uncommon in paediatric patients, occurring at a rate of approximately 4.9 per 100,000. Over half of patients have an associated adnexal mass, most commonly a benign cyst or teratoma [26]. Given that ovarian viability is at stake, there can be limited time for diagnostic work up. Laparoscopy is a reasonable approach for most cases of ovarian torsion [26]. However, in a mass that is at least partially solid, oncologic principles should be maintained, which may require open surgery [4].

## MIS contraindications

Laparoscopic surgery has become the standard of care for operating on benign ovarian masses [27]. However, a laparoscopic approach for dermoid cysts remains controversial due to the possibility of chemical peritonitis and upstaging with spillage [21, 28]. The risk of spillage appears to be more significant with the laparoscopic approach, bilateral cysts and larger masses. While the true risk of chemical peritonitis following cyst spillage remains unknown, some authors recommend an open approach for bilateral dermoid cysts and those larger than 5–8 cm [17, 21]. However, so long as the mass is mobile, others recommend continuing with laparoscopic surgery even for larger masses due to the improved visualisation [4].

Laparoscopic surgery is contraindicated for masses that are known to be malignant or have features highly suspicious for malignancy (≥8 cm, thick walls, loculations or solid components) [10]. This is for several reasons. One, proper staging requires palpation in addition to visual inspection of both ovaries, the omentum and peritoneum [10, 29]. Second, ensuring complete resection is essential to minimising risk for recurrence [30]. Finally, tumour rupture is a feared complication of ovarian surgery as it results in tumour seeding of the abdomen and upstaging of the cancer [31]. There is concern that laparoscopic surgery is associated with increased risk of rupture due to the fact that, unlike in open surgery, MIS requires manipulation of the ovary during the procedure and at the time of extraction [31, 32]. Patients with a ruptured capsule may require adjuvant chemotherapy and experience an estimated increased risk of rate of progression rate of 1.92% and increased rate of all-cause mortality by 1.4%–1.5% [31, 32]. However, literature in paediatric patients has not consistently shown the same association between an MIS approach and tumour rupture, nor inferior outcomes with tumour rupture [22]. Given this ongoing debate, the decision regarding approach should be made on a case-by-case decision with all patient factors considered.

## Surgical approach

### Patient position

Patients should be placed in the supine position or in the lithotomy position if access to the vagina will be required [33, 34]. Steep Trendelenburg is helpful for visualisation, but the patient can be tilted with the head and shoulders left supine if the patient cannot tolerate elevated intracranial pressure [33].

### Trocar sites

Entry via Palmer’s point is recommended if there are significant adhesions, abnormal anatomy or concern about rupture during trocar placement due to the size of the mass [35]. In general, three trocar sites are recommended: a 10–12 mm port through the inferior umbilical fold, a 5 mm suprapubic port located 2 cm above the pubic symphysis and the third port at the counter-McBurney point [16]. Additional ports can be placed in the upper abdomen if extensive debulking is required [18]. The lateral trocars should be inserted under direct visualisation to avoid damage to the inferior epigastric arteries [34].

Single incision laparoscopic surgery (SILS) is an alternative to conventional laparoscopy [36]. As suturing is rarely required, ovarian surgery can be highly suitable for SILS [4]. For cyst removal, SILS has been shown to be safe and effective in adults [37, 38]. Literature in children is limited, but SILS has been successfully used for paediatric ovarian cryopreservation [39].

### Surgical technique

The view of the surgical field differs in children due to the higher upper margin of the bladder, smaller uterus fundus, relatively elongated cervix and smaller ovarian volume [16]. The bowel, other than the sigmoid, should fall away once the patient is placed in Trendelenburg. If additional visualisation is needed, an atraumatic grasper can be used to flip away loops of small bowel [40].

For masses that are thought to be benign, it is still essential to inspect the contralateral ovary and abdominal cavity, including the pouch of Douglas, pelvic and abdominal parietal peritoneum, paracolic gutters, diaphragm, mesentery and small and large bowel [2, 18]. Biopsies and washings should be taken if there are any suspicious findings; however, if the contralateral ovary appears normal on imaging and intraoperatively, it should not be biopsied [18]. Any spillage should be lavaged and documented [41].

The surgical procedure depends on the nature of the mass. Cysts should be aspirated, then the cyst wall resected and removed via an Endo Catch bag [42]. For benign solid neoplasms, OSS is performed by incising the cortex, separating the tumour from the capsule using either blunt or sharp dissection while ensuring the capsule remains intact and removing the tumour [42, 43]. If there is an intraoperative concern for malignancy, a frozen biopsy should be sent in order to inform the decision as to the correct procedure [42]. For malignancies, oophorectomy is performed by incising the peritoneum between the infundibulopelvic (IP) ligament and ureter. Following this, the retroperitoneal space should be dissected to separate the IP ligament from the ureter. The IP ligament, mesovarium and utero-ovarium ligament can then be ligated, and the ovary removed [35, 40].

A surgical pouch should be used to remove the mass or the ovary [16]. If the mass is cystic, it should be placed in the pouch before it is punctured and aspirated [44]. Solid masses may require an additional trocar site or extending an incision. The opening of the bag should be externalised through the incision and the mass manually morcellated before removal [34].

Robotic surgery is increasingly used in adult patients and has been shown to have similar rates of complications and similar oncologic outcomes when compared to open or laparoscopic procedures[45, 18]. The robotic approach for ovarian tumours is less commonly employed in children, and therefore, data are limited [46–48]. One case series described 11 patients, with diagnoses including 6 dermoid tumours, 2 serous papillary cystadenofibromas of the fallopian tube and 3 cystadenomas. An 8 mm optic port and an additional 2–3 accessory ports were used. There were no recurrences or complications with a median follow up of 2.1 years [49]. Alternatively, given the potential for interference between the robotic arms in smaller paediatric abdominal cavities, a single port can be used [50]. One case series describes three patients who underwent robotic OSS for mature teratomas via a 2.5 cm single port site. There was no tumour recurrence during a follow up of 6–18 months [51].

## Tips and pitfalls

There are several key aspects of ovarian surgery. The first priority is ensuring the best chance at successful oncologic outcomes. Therefore, the contralateral ovary needs to be inspected for disease. This is especially true of ovarian teratomas, where contralateral disease occurs in 5%–10% of patients [1, 30, 44]. Additionally, great care must be taken to avoid tumour spillage. For cystic masses, perforation can occur during trocar placement [8]. If intraoperative spillage does occur, copious lavage in reverse Trendelenburg should be performed [8].

Oncologic outcomes need to be balanced against preserving fertility. For patients who present with torsion, the ovary should be given a chance to recover. Even an ovary that appears necrosed intraoperatively can demonstrate blood flow and follicles on future ultrasound [19]. Avoiding biopsy of the contralateral ovary without a clear indication can also help preserve fertility [2].

Additional fertility-preserving techniques should be considered at the time of surgery. For prepubertal children, ovarian stimulation techniques are ineffective, so ovarian tissue cryopreservation (OTC) is the only option [52, 53]. OTC involves the removal of a section of ovarian cortex, freezing and subsequent reimplantation [52]. Data are limited in paediatric patients, with a systematic review of 1,019 children who underwent OTC reporting 18 patients who underwent auto-transplantation and 10 live births [54]. Post-menarche adolescents can also undergo mature oocyte freezing [53].

Finally, there are many critical structures in the pelvis that need to be preserved. Mobilisation of the sigmoid colon can improve visualisation of the left ovary [34]. Ureteral injury occurs in 1%–2% of gynecologic surgeries in adults, so careful attention must be paid to these structures [34].

## Conclusion

Taking a minimally invasive approach for paediatric patients who present with ovarian neoplasms offers the traditional benefits of MIS, as well as fewer adhesions and improved visualisation of pelvic structures, including the contralateral ovary. However, these advantages must be weighed against the need to achieve complete oncologic resection and to minimise the risk of capsule rupture. Additionally, surgeon comfort level may vary with surgeons in some areas constrained by limited access to technical training in MIS. The decision regarding surgical approach should therefore be made on an individualised basis, accounting for all patient factors.

## Conflicts of interest

The authors have no conflicts of interest or funding to declare.

## Funding

There is no funding to disclose.

## References

[1] Sarnacki S, Brisse H (2011). Surgery of ovarian tumors in children. Horm Res Paediatr.

[2] Renaud EJ (2019). Ovarian masses in the child and adolescent: an American Pediatric Surgical Association outcomes and evidence-based practice committee systematic review. J Pediatr Surg.

[3] Trotman GE (2017). Rate of oophorectomy for benign indications in a Children's Hospital: influence of a gynecologist. J Pediatr Adolesc Gynecol.

[4] Fuchs J (2015). The role of minimally invasive surgery in pediatric solid tumors. Pediatr Surg Int.

[5] Seckin B (2011). Laparoscopic treatment of ovarian cysts in adolescents and young adults. J Pediatr Adolesc Gynecol.

[6] Grabowski A, Korlacki W, Pasierbek M (2014). Laparoscopy in elective and emergency management of ovarian pathology in children and adolescents. Wideochir Inne Tech Maloinwazyjne.

[7] Knaus ME (2022). Laparoscopy versus laparotomy for pediatric ovarian dermoids. J Pediatr Surg.

[8] Mosbah A, Nabiel Y, Megahed N (2016). Laparoscopic treatment of ovarian tumors in children: an experience of seven cases in Mansoura University Hospital. Gynecol Surg.

[9] Morowitz M, Huff D, Allmen D (2003). Epithelial ovarian tumors in children: a retrospective analysis. J Pediatr Surg.

[10] Phelps HM, Lovvorn HN (2018). Minimally invasive surgery in pediatric surgical oncology. Children (Basel).

[11] Spinelli C (2012). The role of tumor markers in the surgical approach of ovarian masses in pediatric age: a 10-year study and a literature review. Ann Surg Oncol.

[12] Al Jama FE (2011). Ovarian tumors in children and adolescents--a clinical study of 52 patients in a university hospital. J Pediatr Adolesc Gynecol.

[13] Ray-Coquard I (2018). Non-epithelial ovarian cancer: ESMO clinical practice guidelines for diagnosis, treatment and follow-up. Ann Oncol.

[14] Uzun M (2025). Challenges and innovations in minimally invasive surgery for pediatric patients in Africa: a comprehensive review. Health Sci Rep.

[15] Lawrence AE, Minneci PC, Deans KJ (2019). Ovary-sparing surgery for benign pediatric ovarian masses. Curr Opin Pediatr.

[16] Kim HB (2015). Laparoscopic ovarian surgery in children and adolescents. JSLS.

[17] Piotrowska-Gall A (2024). Laparoscopic ovarian-sparing surgery for the management of benign ovarian lesions in pediatric patients: a retrospective analysis. J Pediatr Surg.

[18] Nezhat FR (2013). Role of minimally invasive surgery in ovarian cancer. J Minim Invasive Gynecol.

[19] Light-Olson H (2023). Minimally invasive adnexa-sparing surgery for benign ovarian and paratubal masses in children. J Pediatr Surg.

[20] Abbas PI (2016). Ovarian-sparing surgery in pediatric benign ovarian tumors. J Pediatr Adolesc Gynecol.

[21] Childress KJ (2017). Intraoperative rupture of ovarian dermoid cysts in the pediatric and adolescent population: should this change your surgical management?. J Pediatr Adolesc Gynecol.

[22] Yousef Y, Pucci V, Emil S (2016). The relationship between intraoperative rupture and recurrence of pediatric ovarian neoplasms: preliminary observations. J Pediatr Adolesc Gynecol.

[23] Galazka P (2019). Minimally invasive surgery in pediatric oncology: proposal of guidelines. Anticancer Res.

[24] Corrado G (2015). Laparoscopic debulking surgery in the management of advanced ovarian cancer after neoadjuvant chemotherapy. Int J Gynecol Cancer.

[25] Tozzi R (2024). Feasibility of laparoscopic Visceral-Peritoneal Debulking (L-VPD) in patients with stage III-IV ovarian cancer: the ULTRA-LAP trial pilot study. J Gynecol Oncol.

[26] Nguyen KP (2022). Ovarian torsion: presentation and management in a pediatric patient. Case Rep Obstet Gynecol.

[27] Ergun-Longmire B, Greydanus DE (2024). Ovarian tumors in the pediatric population: an update. Dis Mon.

[28] von Allmen D (2005). Malignant lesions of the ovary in childhood. Semin Pediatr Surg.

[29] Oltmann SC (2010). Pediatric ovarian malignancies: how efficacious are current staging practices?. J Pediatr Surg.

[30] Terenziani M (2015). Mature and immature teratoma: a report from the second Italian pediatric study. Pediatr Blood Cancer.

[31] Dioun S (2021). Intraoperative rupture of the ovarian capsule in early-stage ovarian cancer: a meta-analysis. Obstet Gynecol.

[32] Matsuo K (2020). Minimally invasive surgery and risk of capsule rupture for women with early-stage ovarian cancer. JAMA Oncol.

[33] Xiao JS (2021). Laparoscopic gynaecological surgery in the context of maintaining normal intracranial pressure. BMJ Case Rep.

[34] Lozada Y, Bhagavath B (2017). A review of laparoscopic salpingo-oophorectomy: technique and perioperative considerations. J Minim Invasive Gynecol.

[35] Lawson AA (2022). Oophorectomy.

[36] Misawa A (2023). Successful use of single-port laparoscopic surgery for ovarian cyst removal during pregnancy: a case series of three cases. J Surg Case Rep.

[37] Wang X, Li Y (2021). Comparison of perioperative outcomes of single-port laparoscopy, three-port laparoscopy and conventional laparotomy in removing giant ovarian cysts larger than 15 cm. BMC Surg.

[38] Schmitt A (2024). The effects of a laparoscopy by single-port endoscopic access in benign adnexal surgery: a randomized controlled trial. J Minim Invasive Gynecol.

[39] Metcalfe K (2022). Conventional 3-port vs. single-incision laparoscopic oophorectomy for ovarian cryopreservation in paediatric surgery: a retrospective case-note review. Ann Pediatr Surg.

[40] Ellison E (2022). Zollinger’s Atlas of Surgical Operations.

[41] Braungart S (2022). Standardizing the surgical management of benign ovarian tumors in children and adolescents: a best practice Delphi consensus statement. Pediatr Blood Cancer.

[42] Raźnikiewicz A, Korlacki W, Grabowski A (2020). The role of laparoscopy in paediatric and adolescent gynaecology. Wideochir Inne Tech Maloinwazyjne.

[43] Khai T (2023). Characteristics and outcomes of pediatric ovarian germ cell tumors: a report of 162 cases. Biomed Res Ther.

[44] Tatekawa Y (2021). Surgical approach to three pediatric patients with ovarian tumors: laparoscopic excision using a specimen retrieval bag. J Surg Case Rep.

[45] Park J (2023). Robotic surgery in gynecology: the present and the future. Obstet Gynecol Sci.

[46] Gallotta V (2023). Robotic surgery in ovarian cancer. Best Pract Res Clin Obstet Gynaecol.

[47] Pelizzo G, Nakib G, Calcaterra V (2019). Pediatric and adolescent gynecology: treatment perspectives in minimally invasive surgery. Pediatr Rep.

[48] Esposito C (2024). Robotic-assisted surgery for gynecological indications in children and adolescents: European multicenter report. J Robot Surg.

[49] Vatta F (2021). Robotics-assisted pediatric oncology surgery-a preliminary single-center report and a systematic review of published studies. Front Pediatr.

[50] Xie XX (2019). Robotic-assisted resection of ovarian tumors in children: a case report and review of literature. World J Clin Cases.

[51] Xu D (2022). Ensuring safety and feasibility for resection of pediatric benign ovarian tumors by single-port robot-assisted laparoscopic surgery using the da Vinci Xi system. Front Surg.

[52] Dinikina Y (2021). Ovarian tissue cryopreservation in prepubertal patients with oncological diseases: multidisciplinary approach and outcomes. J Matern Fetal Neonatal Med.

[53] Dolmans MM (2021). Fertility preservation: how to preserve ovarian function in children, adolescents and adults. J Clin Med.

[54] Corkum KS (2019). Fertility and hormone preservation and restoration for female children and adolescents receiving gonadotoxic cancer treatments: a systematic review. J Pediatr Surg.

